# Progress in Obstetrics

**Published:** 1895-11-23

**Authors:** 


### Progress in Obstetrics.

(*Continued from page* 102.)

**The Preservation of the Perinæum.**—Dr. McCartie[9] suggests a new method to avoid rupture of the perinæum. It is the most important work of the physician in the end of the second part of labour to devote his attention to the preservation of this tissue; he can do very little to help the uterine force, but he can do much to control its action on the lower genital segment, by preventing the child's head from coming in contact too violently or too soon with a tissue which may not be prepared to allow the passage of the fœtal head. The old plan was to "support the perinæum," meaning that the hand should be placed against the bulging perinæum to prevent it bulging too much. This only increases the uterine contractions, and may even retard the labour, since the pressure acts too directly in the axis of the propelling force. It is the marginal edge which tears, and by the above means only the central portion of the perinæum is supported, and this really needs the least support. Other old methods entail a double procedure—first, they aim at preventing the too rapid advance of the head; and, secondly, they make use of an artificial propelling force, by which the head is pressed toward the outlet under the pubic arch. One must necessarily contend against the too rapid advancement of the head, as it is this spasmodic force from behind which ruptures the perinæum. The second part of the procedure seems worthy of some modifications—if, indeed, we should attempt to use any force at all in this direction—with the view of pushing the fœtal head through the vulvar outlet. It is easy in a large multipara, if it is necessary to deliver quickly, to push forward the head from behind, but it is very questionable in a primipara if this proceeding is justifiable, as I believe it often helps to lacerate the very parts which we are seeking to preserve. This action on the part of the physician causes the head to extend itself forward and sweep over the perinæal floor. Dr. McCartie advocates the exact opposite of this; instead of seeking to extend the head, our aim should be to try and keep it in a constant state of flexion. When the occiput has reached the outlet it should then be grasped and the head kept flexed, and this will enable the occiput to slip from under the pubic arch earlier than it otherwise would, and also helps to dilate the perinæum by keeping the forehead region rigidly compressed against it. When a part of the occipital region is born it should be grasped in front by the fingers of the right hand and the thumb behind on some portion of the parietal bone. By this method the head can be kept flexed in a most easy and satisfactory manner. By this means also the uterine force can be more easily counteracted and the efforts of the patient more easily controlled, because the head being thoroughly flexed the force is controlled in the direct axis of the uterus; and, more important than this, the elasticity of the perinæum is counteracted, and extension over its floor is prevented. Extension of the head over the perinæum as it passes forward is not the natural process of birth; it is a convenient way to precipitate the birth of the child, but it is premature and unnecessary when the perinæum is rigid and liable to rupture. The occiput in its first passage through the vulva never tears it, it gradually thins and stretches the soft parts until it slips under the pubic arch. Dr. McCartie's main suggestion then is that flexion should be maintained from first to last, and he then maintains that rupture of the perinæum will be a very rare event.

**Rare Form of Post-Partum Hæmorrhage.**—Dr. R. A. Gibbons[10] has published a very interesting article on tonic spasm of the body of the uterus as a cause of post-partum hæmorrhage. The condition is so very rare that it is quite possible that many practical obstetricians doubt its existence at all. From the external appearance of the uterus in these cases one would be led to suppose that it was in a state of moderately firm contraction, but an internal examination shows that it is simply a tonic spasm of its walls. Dr. Leahy[11] records two such cases that have occurred in his own practice, and relates how very serious was the hæmorrhage in each, although apparently the uterus was externally moderately firmly contracted—the body being felt above the pubes in each case perfectly hard, but not regularly contracted. He suggests the remedy for this state of things would be to give chloroform and so relax the tonic spasm.

**The Care of the Breasts after Labour.**—Dr. Edward Reynolds[13] states that our treatment should be such that the patient shall be spared the pain and other evil consequences which follow an undue engorgement of the breasts. To this end we have the following expedients with which to work: The baby, the breast-pump, massage, the bandage, collodion dressing, poultice, ice-cap, and the ice-poultice.

The baby should be used only when the stimulating effect of nursing can be endured, to continue the supply of milk, and when nothing harmful to the child is in the milk. The breast-pump should never be used to such an extent as to cause pain, for it only withdraws the milk in the large ducts near the nipple, and it should be used as an adjunct to massage. Massage decreases the sensitiveness of the breast, and also empties the milk ducts; it should be practised with a lubricant with the greatest possible gentleness, using a stroking movement from the periphery to the centre. The process may be continued until deep stroking movements are made, provided it does not cause any pain.

The breast bandage is used (1) to give surgical rest in all cases where the breast is affected; (2) to give support to a pendulous breast when the weight will lead to engorgement; (3) as a means of applying pressure and keeping the breast from filling. The best form of bandage is a T-shaped piece of cloth, the tail of the T is passed under the patient, across the scapular region, the ends of the cross-bar are brought together (one above and the other below the breasts).

Collodion is very distressing to the patient, and if applied must include the whole breast tissue.

The hot poultice is used for the relief of symptoms attending a distinct phlegmon, when we have given up all hope of its resolving.

Cold dressings are used for checking inflammatory affections in their inception, and before pus appears. Cold may be applied in several ways: (1) Small tubes made into a spiral through which cold water continuously flows may be applied to the affected breast; (2) rubber ice-bag with ice so finely divided as not to irritate the breast; (3) the ice-poultice is prepared from bran, with small fragments of ice scattered through it; as the ice melts the bran absorbs the water, and also prevents the ice from irritating the sensitive breast. There should be enough to prevent dripping, but still plenty of ice well distributed amongst it.

If an abscess should threaten, due to inspissation of the milk within the ducts and acini of a given lobe of the gland from infection through the nipple, then massage, rest, and a bandage will generally remove it in from twenty to forty-eight hours. If, on the other hand, the connective tissue is the part affected, accompanied by pyrexia and reddening of the skin, then massage must not be employed, but the case must be treated on strictly antiseptic surgical principles.

[9] N.Y. Med. Journ., May 25, 1895. [10] Lancet. Aug. 3, 1895. [11] Comptes-Rendus du Congrès Périodique Internat. de Gynécol. et d'Obstét. [13] Amer. Med. Surg. Bulletin, June 15, 1895. [13] Boston Med. and Surg. Journ., CXXXII., No. 15, p. 345.

